# Phloem: the integrative avenue for resource distribution, signaling, and defense

**DOI:** 10.3389/fpls.2013.00471

**Published:** 2013-11-25

**Authors:** Aart J. E. van Bel, Ykä Helariutta, Gary A. Thompson, Jurriaan Ton, Sylvie Dinant, Biao Ding, John W. Patrick

**Affiliations:** ^1^Department of Biology, Institute of General Botany, Justus-Liebig-UniversityGiessen, Germany; ^2^Plant Molecular Biology Lab, Institute of Biotechnology, University of HelsinkiHelsinki, Finland; ^3^Department of Plant Science, College of Agricultural Sciences, The Pennsylvania State University, University ParkPA, USA; ^4^Department of Animal and Plant Sciences, University of SheffieldSheffield, UK; ^5^Institut Jean-Pierre Bourgin UMR1318 INRA-AgroParisTech, Institut National de la Recherche AgronomiqueVersailles, France; ^6^Department of Molecular Genetics, The Ohio State UniversityColumbus, OH, USA; ^7^School of Environmental and Life Sciences, The University of NewcastleCallaghan, NSW, Australia

**Keywords:** phloem development, phloem physiology, phloem transport, phloem-located resistance, phloem-mobile signaling, phloem structure, phloem cell biology, electropotential waves

Research over the past 20 years has revealed new functions of the phloem beyond resource allocation to a system that combines distribution and messaging (Thompson and van Bel, 2013) analogous to the circulatory and nervous systems in animals. Apart from allocating resources for maintenance and growth, the phloem distributes hormonal signals and a broad spectrum of protein- and RNA-based messages throughout the plant to regulate a myriad of physiological and developmental processes. Resources and signals, collectively, coordinate development, and growth as well as integrate responses to both biotic and abiotic environmental challenges. The transport of organic compounds such as sugars, amino acids, and lipidic substances through the phloem are exploited by a vast and diverse range of pathogens such as viruses, fungi, nematodes, aphids, and other phloem-feeding insects. Given its ubiquitous occurrence in land plants, the phloem seems to be the integrative tissue *par excellence*. The contributions included in this research topic encompass the entire bandwidth of known phloem functions with emphasis on the diversity in structures and functions.

Differentiation and development of vascular cells is complex and not well understood. Vascular development partly depends on environmental cues that have also impacted the evolution of vascular systems and phloem transport mechanisms. A novel phylogenetic approach to identify genes involved in vascular development (Martinez-Navarro et al., 2013) shows that several vascular genes are expressed in green algae (Chlorophyta), the ancestors of land plants. Analysis of vascular genes in non-vascular and ancient vascular plants indicates that coordinated expression of gene sets led to the emergence of the present vascular system (Martinez-Navarro et al., 2013). Representatives of the Dof gene family are among the transcription factors involved in vascular differentiation (Le Hir and Bellini, 2013). Nematode saliva has the remarkable ability to induce re-differentiation of phloem cells and their neighbors with the objective to “tap” the sieve-tube sap (Absmanner et al., 2013).

Ample attention has been paid to the structural diversity, particularly in the phloem-loading zone, where environmental changes have unbuffered, and profound effects. The impact of diverse environmental conditions on leaf structure and carbohydrate processing is demonstrated with *Arabidopsis* ecotypes (Adams et al., 2013; Cohu et al., 2013a,b). Within the Asteridae, there is an immense diversity in minor-vein structures and companion cells (Batashev et al., 2013), which promises a higher variety of phloem-loading modes in dicots than previously suspected (Slewinski et al., 2013). It appears that a strict subdivision between apoplasmic and symplasmic phloem-loading species must be abandoned, since many species dispose over the devices to operate both modes in parallel. A structural feature of apoplasmic phloem loading in dicotyledons—the involvement of transfer cells—is highlighted in two contributions: one on the evolutionary trends, function and induction (Andriunas et al., 2013) and the other on transcriptional regulators of cell wall invagination (Chinnappa et al., 2013). A physiological feature of “active” symplasmic phloem loading is the size-selective transfer of sugars through plasmodesmata, which is challenged here using mathematical parameters (Liesche and Schulz, 2013). In grasses, the arrangement and ultrastructure of collection phloem suggest an apoplasmic mode of phloem loading (Botha, 2013; Slewinski et al., 2013). However, the functions of two principal structures in monocotyledonous leaves i.e., thick-walled sieve tubes and transverse veins remain puzzling (Botha, 2013). Thick-walled sieve tubes may be viewed as transformed phloem parenchyma cells (Slewinski et al., 2013) engaged in temporary storage (Botha, 2013).

Carbohydrate processing may be more homogeneous in transport phloem (Slewinski et al., 2013) than in the phloem-loading zone. Yet, permanently changing conditions require flexible and diverse solutions for release/retrieval along the pathway (De Schepper et al., 2013). Central to the release/retrieval concept is the intercellular competition for sugars that is revealed with electrical methods for *in situ* measurement of sucrose uptake parameters (Hafke et al., 2013). The strong influence of environmental impacts on phloem functioning at each level is addressed in one comprehensive review by Lemoine et al. (2013).

Long-distance signaling via the phloem can be accomplished by a variety of physiological mechanisms. Electrical signaling along sieve tubes affects both the physiology of distant leaves as well as photosynthate release/retrieval (Fromm et al., 2013). Long-distance effects of hormonal signaling on plant development have been demonstrated by expressing melon Aux/IAA genes into tomato plants (Golan et al., 2013). The translocation of microRNAs through the phloem regulates and coordinates distant processes such as mineral homeostasis (Kehr, 2013). Evidence is emerging for remote control of developmental processes in roots and tubers by phloem-mobile mRNAs (Hannapel et al., 2013). However, providing unambiguous evidence for some of the key processes implicated remains challenging and is open for debate (Hannapel, 2013; Suarez-Lopez, 2013).

RNA-species are most likely translocated as complexes with RNA-binding proteins (Pallas and Gomez, 2013). As RNAs, several viruses are translocated as ribonucleoprotein complexes (Hipper et al., 2013) which might protect the viral core against the adverse sieve-tube environment and/or confer tagging for invasion of specific target cells. While the viral complexes move through sieve tubes with the mass flow, phytoplasmas with sizes exceeding the diameters of the sieve pores—that are occluded anyway in response to infection—may be disseminated by alternative mechanisms. Remarkably, some plants species are able to overcome phytoplasma infection through the so-called “recovery reactions” that are mediated through callose degradation in sieve tubes (Santi et al., 2013).

The phloem contains attractants and repellents for animal pathogens. Present work indicates that sterols serve as attractants (Behmer et al., 2013) to phloem-feeding insects that have a deficient sterol synthesis, whereas benzylisoquinoline alkaloids serve as lethal repellents (Lee et al., 2013). It appears that phloem cells produce and transport an arsenal of defense compounds. The location of these anti-insect chemicals is significant e.g., for genetic manipulation of plants. Defense compounds against aphids may reside either or both in the pre-phloem pathway or inside the phloem cells themselves (Will et al., 2013). It is postulated that the major function of the extrafascicular phloem in cucurbits is to combat insects (Gaupels and Ghirardo, 2013). In legumes, giant proteins bodies (forisomes) may be involved in plant defense by rapid sieve-pore occlusion in response to an aphid attack (Jekat et al., 2013). The interactions between plants and aphids appear extremely complex (Louis and Shah, 2013) which hinders the development of molecular strategies for insect control. Nonetheless, strategies are being developed to increase the plant resistance against aphids (Will et al., 2013), and in one case by engineering of RFO phloem loading (Cao et al., 2013). The strong increment of RFOs in the sieve-tubes rendered the plants less attractive to aphids.

As with other research fields, phloem research reveals an ever receding horizon with undreamed possibilities. This topic shows the amazingly diverse ability of plants to cope with an infinite number of environmental challenges by virtue of the vascular tissues.

## References

[B1] Absmanner B. Stadler R. Hammes U. Z. (2013). Phloem development in nematode-induced feeding site; the implications of auxin and cytokinin. Front. Plant Sci. 8:241 10.3389/fpls.2013.00241 23847644 PMC3703529

[B2] Adams W. W. Cohu C. M. Muller O. Demmig-Adams B. (2013). Foliar phloem infrastructure in support of photosynthesis. Front. Plant Sci. 4:194 10.3389/fpls.2013.00194 23785375 PMC3682110

[B3] Andriunas F. Zhang H. Xia X. Patrick J. W. Offler C. E. (2013). Intersection of transfer cells with phloem biology—broad evolutionary trends, function, and induction. Front. Plant Sci. 4:221 10.3389/fpls.2013.00221 23847631 PMC3696738

[B4] Batashev D. R. Pakhomova M. V. Razumovskaya A. V. Voitsekhovskaja O. V. Gamalei Y. V. (2013). Cytology of the minor-vein phloem in 320 species from the subclass Asteridae suggests a high diversity of phloem-loading modes. Front. Plant Sci. 4:312 10.3389/fpls.2013.00312 23970890 PMC3748319

[B5] Behmer S. Olszewski N. Sebastiani J. Palka S. Sparacino G. Grebenok R. J. (2013). Plant phloem sterol contents: forms, putative functions, and implications for phloem-feeding insects. Front. Plant Sci. 4:370 10.3389/fpls.2013.00370 24069026 PMC3781331

[B6] Botha T. (2013). A tale of two neglected systems—structure and function of the thin- and thick-walled sieve tubes in monocotyledonous leaves. Front. Plant Sci. 4:297 10.3389/fpls.2013.00297 23964280 PMC3734358

[B7] Cao T. Lahiri I. Singh V. Louis J. Shah J. Ayre B. G. (2013). Metabolic engineering of raffinose-family oligosaccharides in the phloem reveals alteration in carbon partitioning and enhances resistance to green peach aphid. Front. Plant Sci. 4:263 10.3389/fpls.2013.00263 23882277 PMC3715723

[B8] Chinnappa K. S. Nguyen T. T. Hou J. Wu Y. McCurdy D. W. (2013). Phloem parenchyma transfer cells in Arabidopsis – an experimental system to identify transcriptional regulators of wall ingrowth formation. Front. Plant Sci. 4:102 10.3389/fpls.2013.00102 23630536 PMC3634129

[B9] Cohu C. M. Muller O. Demmig-Adams B. Adams W. W. (2013a). Minor loading vein acclimation for three Arabidopsis thaliana ecotypes in response to growth under different temperature and light regimes. Front. Plant Sci. 4:240. 10.3389/fpls.2013.00240 23847643 PMC3701806

[B10] Cohu C. M. Muller O. Stewart J. J. Demmig-Adams B. Adams W. W. (2013b). Association between minor loading vein architecture and light- and CO_2_-saturated rates of photosynthetic oxygen evolution among Arabidopsis thaliana ecotypes from different latitudes. Front. Plant Sci. 4:264 10.3389/fpls.2013.00264 23898338 PMC3724126

[B11] De Schepper V. Bühler J. Thorpe M. Roeb G. Huber G. van Dusschoten D. (2013). ^11^C-PET imaging reveals transport dynamics and sectorial plasticity of oak phloem after girdling. Front. Plant Sci. 4:200 10.3389/fpls.2013.00200 23785380 PMC3684848

[B12] Fromm J. Hajirezaei M.-R. Becker V. K. Lautner S. (2013). Electrical signaling along the phloem and its physiological responses in the maize leaf. Front. Plant Sci. 4:239 10.3389/fpls.2013.00239 23847642 PMC3701874

[B13] Gaupels F. Ghirardo A. (2013). The extrafascicular phloem is made for fighting. Front. Plant Sci. 4:187 10.3389/fpls.2013.00187 23781225 PMC3678090

[B14] Golan G. Betzer R. Wolf S. (2013). Phloem-specific expression of a melon Aux/IAA in tomato plants alters auxin sensitivity and plant development. Front. Plant Sci. 4:329 10.3389/fpls.2013.00329 23986770 PMC3750518

[B15] Hafke J. B. Höll S.-R. Kühn C. van Bel A. J. E. (2013). Electrophysiological approach to determine kinetic parameters of sucrose uptake by single sieve elements or phloem parenchyma cells in intact Vicia faba plants. Front. Plant Sci. 4:274 10.3389/fpls.2013.00274 23914194 PMC3728481

[B16] Hannapel D. J. (2013). A perspective on photoperiodic phloem-mobile signals that control development. Front. Plant Sci. 4:295 10.3389/fpls.2013.00295 23935603 PMC3731531

[B17] Hannapel D. J. Sharma P. Lin T. (2013). Phloem-mobile RNAs and root development. Front. Plant Sci. 4:257 10.3389/fpls.2013.00257 23882275 PMC3713340

[B18] Hipper C. Brault V. Ziegeler-Graf V. Revers F. (2013). Viral and cellular factors involved in phloem transport of plant viruses. Front. Plant Sci. 4:154 10.3389/fpls.2013.00154 23745125 PMC3662875

[B19] Jekat S. B. Ernst A. M. von Bohl A. Zielonka S. Twyman R. M. Noll G. A. (2013). P-proteins in Arabidopsis are heteromeric structures involved in rapid sieve tube sealing. Front. Plant Sci. 4:225 10.3389/fpls.2013.00225 23840197 PMC3700381

[B20] Kehr J. (2013). Systemic regulation of mineral homeostasis by micro RNAs. Front. Plant Sci. 4:145 10.3389/fpls.2013.00145 23720667 PMC3655319

[B21] Lee E.-J. Hagel J. M. Facchini P. J. (2013). Role of the phloem in the biochemistry and ecophysiology of benzylisoquinoline alkaloid metabolism. Front. Plant Sci. 4:182 10.3389/fpls.2013.00182 23781223 PMC3678098

[B22] Le Hir R. Bellini C. (2013). The plant-specific Dof transcription factors family: new players involved in vascular system development and functioning in Arabidopsis. Front. Plant Sci. 4:164 10.3389/fpls.2013.00164 23755058 PMC3665933

[B23] Lemoine R. La Camera S. Atanassova R. Dédaldéchamps F. Allario T. Pourtau N. (2013). Source-to-sink transport of sugar and regulation by environmental factors. Front. Plant Sci. 4:272 10.3389/fpls.2013.00272 23898339 PMC3721551

[B24] Liesche J. Schulz A. (2013). SModeling the parameters for plasmodesmal sugar filtering in active symplasmic phloem loaders. Front. Plant Sci. 4:207 10.3389/fpls.2013.00207 23802006 PMC3685819

[B25] Louis J. Shah J. (2013). Arabidopsis thaliana—Myzus persicae interaction: shaping the understanding of plant defense against phloem-feeding aphids. Front. Plant Sci. 4:213 10.3389/fpls.2013.00213 23847627 PMC3696735

[B26] Martinez-Navarro A. C. Xoconostle-Cazares B. Galvan-Gordillo S. V. Ruiz-Medrano R. (2013). Vascular gene expression: a hypothesis. Front. Plant Sci. 4:261 10.3389/fpls.2013.00261 23882276 PMC3713349

[B27] Pallas V. Gomez G. (2013). Phloem RNA-binding proteins as potential components of the long-distance RNA transport system. Front. Plant Sci. 4:130 10.3389/fpls.2013.00130 23675378 PMC3650515

[B28] Santi S. De Marco F. Polizzotto R. Grisan S. Musetti R. (2013). Recovery from stolbur disease in grapevine involves changes in sugar transport and metabolism. Front. Plant Sci. 4:171 10.3389/fpls.2013.00171 23761800 PMC3671194

[B29] Slewinski T. L. Zhang C. Turgeon R. (2013). Structural and functional heterogeneity in phloem loading and transport. Front. Plant Sci. 4:244 10.3389/fpls.2013.00244 23847646 PMC3701861

[B30] Suarez-Lopez P. (2013). A critical appraisal of phloem-mobile signals involved in tuber induction. Front. Plant Sci. 4:253 10.3389/fpls.2013.00253 23882274 PMC3712254

[B31] Thompson G. A. van Bel A. J. E. (2013). Phloem. Molecular Cell Biology, Systemic Communication, Biotic Interactions. Cichester: Wiley-Blackwell ISBN-13: 978-0-8138-0349-4/2012.

[B32] Will T. Fuchs A. C. Zimmermann M. R. (2013). How phloem-feeding insects face the challenge of phloem-located defenses. Front. Plant Sci. 4:336 10.3389/fpls.2013.00336 24009620 PMC3756233

